# A bibliometric analysis of international publications and citation trends of articles in mental health produced by Chinese institutions in mainland China (1990–2019)

**DOI:** 10.1017/gmh.2021.35

**Published:** 2021-09-28

**Authors:** Francesca Severino, Valeria Scotti, Tianhong Zhang, Yuchen Zheng, Annalisa De Silvestri

**Affiliations:** 1 Renji Hospital, Clinical Research Center, Shanghai Jiao Tong University School of Medicine, Shanghai, China; 2 Center for Scientific Documentation of IRCCS Policlinico San Matteo Foundation, Pavia, Italy; 3 Shanghai Mental Health Center, Shanghai Jiao Tong University School of Medicine, Shanghai, China; 4 Clinical Epidemiology and Biometric Unit, Foundation IRCCS, Policlinico San Matteo, Pavia, Italy

**Keywords:** Bibliometric analysis, China, global mental health, mental health research

## Abstract

**Background:**

The recognition of the importance of mental health as a health-target to be pursued at a global level has received additional theoretical legitimacy through its inclusion in the United Nations (UN) 2030 Agenda for Sustainable development. The theoretical axiom – mental health as a development priority – is today expected to drive the focus of research efforts and orient the future policies and funds expenditures, at global and local level. According to these premises, it becomes central to track the international trajectories of mental health research and how the different countries are progressively defining their role in the global mental health effort. In this paper we have focused on China. In light of heavy burden of mental and substance use disorders affecting this country, and considering the impact of this burden at a global level, a basic research was conducted with the main aim of offering a preliminary view on the Chinese scientific activity within the context of global mental health research. This study is not intended to assess the quality of Chinese research, but merely to retrieve and measure a specific output of this research: the articles in mental-health produced by Chinese institutions based in mainland China, published in international journals. Although the publication of articles in internationally indexed journals in not exhaustive of China's scientific activity in global mental health, it is nevertheless informative of the production of new knowledge, it allows an assessment of the impact of this knowledge at the global scientific community level and it could partially reflect the Chinese capacity to benefit from research conducted globally.

**Objective:**

In consideration of the very limited number of studies assessing the collective evidence of Chinese research in mental health, we developed our analysis with the purpose of providing a preliminary picture of the Chinese contribution, in terms of scientific publications, in this field of knowledge. Our research performs a bibliometric analysis on the articles in mental-health produced by Chinese institutions based in mainland China and published in English-language SCI-E and SSCI journals from 1990 to 2019, providing a measure of the impact of this research at the global scientific community level.

**Methods:**

We performed a search on the Web of Science (WoS) using seven mental and substance use disorders according to their global prevalence, as per estimates of the Global Burden of Disease 2019. A dataset including the overall number of publications for seven diseases was created and exported in InCites. The dataset was analysed on the basis of 11 research areas (WoS categories) to which mental health topic is associated in SCI-E and SSCI journals in WoS. We further extracted publications that originated in mainland China. The citational trends over time are calculated with nonparametric test for trends across ordered groups. An evaluation of the impact of the Chinese scientific production is provided by the number of citations received at the global scientific community level, both as average and percentile.

**Results:**

From 1990 to 2020 the overall Chinese scientific production in mental health has been generally increasing, reaching the highest growth in the last decade. A statistically significant increase (*p* < 0.001) is reported for articles produced by Chinese institutions in mainland China regarding ‘depression*’, ‘bipolar disorders*’ and ‘schizophrenia*’. Published Chinese research is mostly included in SCI-E journals. There is a substantial overlap regarding the average number of citations for articles in mental-health produced by Chinese institutions and the rest of the world. Despite the increasing trend, the percentage of articles in mental health produced by Chinese institutions in mainland China on the overall scientific production worldwide is below 10%.

**Conclusion:**

Notwithstanding a substantial increase in the last decade, the volume of Chinese publications appears to be very limited, thus resulting in a relatively low impact at a global level. These results are affecting the potential contribution of Chinese research in the global mental health effort.

## Introduction

The inclusion of mental health within the United Nations programmatic lines for a sustainable development in 2015 has marked at least two important and interdependent changes. First, by connecting mental health and development, the mental health research paradigm widened its epistemological coordinates from *global health priority* to *development priority*. A new trajectory of research was conceptualised, in which the ideas of development and mental health need to be rethought and carefully connected, locally and globally. Second, it has been agreed that mental health must be given a priority within policies and funding at national and international levels, foreseeing a re-prioritisation of actions and a multisectoral research to guide their implementation. The UN 2030 Agenda for Sustainable development states that ‘all countries and all stakeholders, acting in collaborative partnership, are now committed to the prevention and treatment of non-communicable diseases, including behavioural, developmental and neurological disorders, which constitute a major challenge for sustainable development’ (United Nations, 2015). China has committed to fulfil the UN Sustainable Development Goals (SDGs) agenda in its ‘Healthy China 2030’ blueprint (Tan *et al*., 2017). However, according to the systematic analysis of the current situation and progress toward the 2030 health-related SDGs performed by Chen S and colleagues in 2019 and the recent UN-ESCAP progress report on SDG in Asia and Pacific (United Nations, Economic and Social Commission for Asia and the Pacific, 2021), China appears no longer on track to meet many of its expected health targets. Chen *et al*. (2019) are reporting that, despite the disease burden caused by mental disorders represents a critical issue for China, the health system response seems to be inadequate. The critical issues highlighted are an insufficient budget compounded by stigma, low rates of case notification, diagnosis and treatment (especially for non-severe mental disorders), low quality of mental health services and a weak capacity of the primary care system (Chen *et al*., 2019).

With reference to Goal 3 good health and well-being, the UN-ESCAP progress report 2021 states that most of the progress in East and North-East Asia is due to early achievement of the targets related to maternal, under-5 and neonatal mortality. Otherwise, ‘Non-Communicable Disorders (NCD) and mental health’ is one of the sub-targets that requires, in order to be achieved, a strengthening of efforts to accelerate its progress towards the expected SDG threshold.

The importance of research, as part of the public health response to address the burden of mental illness, have been emphasised as essential to map mental health needs, propose cost-effective and culturally appropriate interventions, monitor the process of their implementation, and explore the obstacles that prevent recommended strategies from being implemented (Zhang *et al*., 1998; Saraceno and Saxena, 2004; Maj, 2005; Lancet Mental Health Group, 2007; Patel, 2007; Patel and Kim, 2007; Saxena *et al*., 2007; Global Forum for Health Research, 2008; Huang, 2008; Razzouk *et al*., 2010). The volume of published research in mental health, although not exhaustive of a country's scientific activity in that field of knowledge, could partially inform on the efforts made at a local level in providing new evidences and, alongside, on the capacity to include and benefit from research conducted globally (Paraje *et al*., 2005). To date, a very limited number of studies have attempted to analyse the amount of Chinese mental-health related publications in international journals. In this paper we will attempt to quantify the scientific contribution of Chinese research institutions by assessing their internationally published articles.

**Fig. 1.**
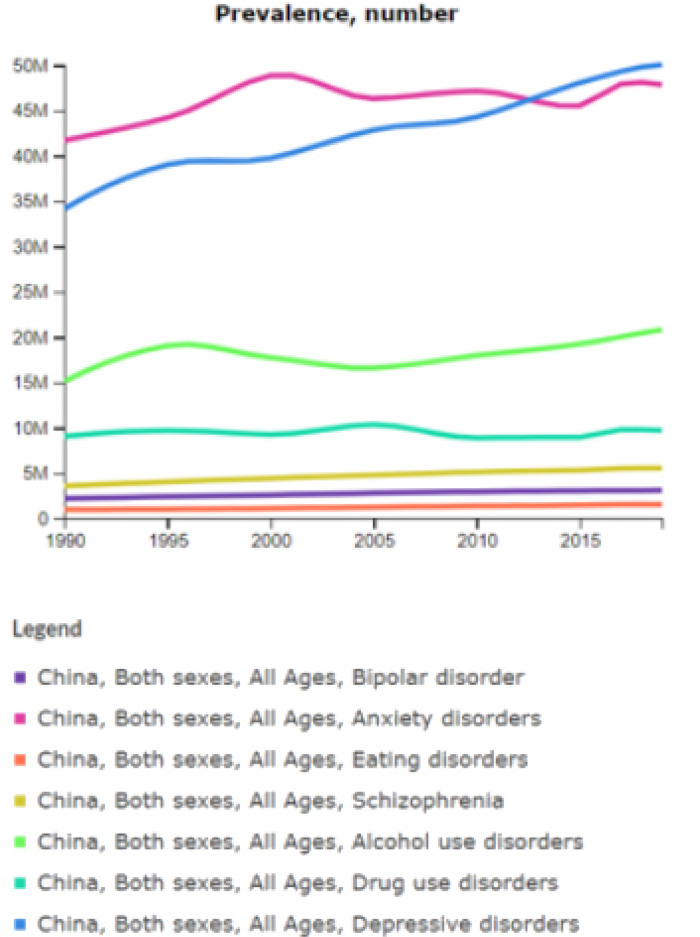
Prevalence by mental and substance use disorders, Global and China, Source: IHME, Global Burden of Disease 1999, 2009, 2019.

According to the estimates of the Global Burden of Disease (GBD) 2019 (Global Burden of Disease Study 2019, 2020), the most prevalent disorders in China are ‘depressive disorders’, ‘anxiety’ and ‘alcohol use disorders’ accounting for, respectively, 3.76% (50 million), 3.51% (48 million) and 1.53% (21 million) of the overall population (1.4 billion). Followed by ‘drug use disorders’, ‘schizophrenia’, ‘bipolar disorders’ and ‘eating disorders’ (Global Health Data Exchange, 2020). The prevalence for the world and China, for each disorder, is synthetised in Fig. 1.

To perform our study, we selected these seven specific disorders (depressive disorders, anxiety, alcohol use disorders, drug use disorders, schizophrenia, bipolar disorders and eating disorders) and launched a search by topic on the WoS core collection database. We calculated the total number of articles in mental-health produced by Chinese institutions in mainland China and published in international SCIE/SCI journals and we finally evaluated their impact at the global scientific community level.

## Methods

To quantify the overall volume of the Chinese scientific production in mental health, covering articles in a broad array of scientific, medical, and social sciences journals, we have chosen ‘Web of Science’ (WoS), limiting our search to two core collection databases: the Science Citation Index Expanded (SCI-E) and the Social Sciences Citation Index (SSCI). The rationale behind favouring WoS over other databases aligns with the objectives of the current research. The decision to examine scientific publications over a 30-year period (1990–2019) led us to choose WoS – which, unlike other databases, provides information that can be dated back to the 1900s. Additionally, WoS core collection represents the data source for InCites Benchmarking and Analytics, a customised web-based research evaluation tool. In executing our search, we used standard datasets created in WoS and then we exported them in InCites. To perform normalised metrics, it is mandatory to calculate consistent and accurate baselines. As we completed our bibliometric analysis, the choice of using WoS and InCites represented a further guarantee of baseline consistency, as during the process of datasets extraction from the WoS and exportation in InCites, the citation counts remained unchanged.

The general WoS platform allows to retrieve articles produced in different research areas, but among the 254 available, there is no thematic area specific to mental health. Therefore, in developing the methodology, we decided to search for articles in mental health using an approach based on specific diseases. In accordance with the estimates from the Global burden of disease (1999, 2009 and 2019) and data on the prevalence of mental and substance use disorders in China, we selected seven specific disorders: depressive disorders, anxiety, alcohol use disorders, drug use disorders, schizophrenia, bipolar disorders and eating disorders (Global Burden of Disease Study 2019, 2020).

**Fig. 2.**
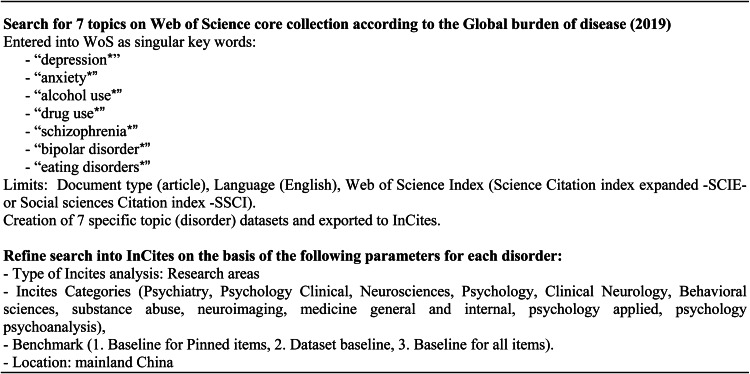
Search methodology in Web of Science and InCites.

For each of these disorders, we launched a search by topic, in June 2020, on the WoS core collection database customising a 10 years range per extraction, from 1990 to 2019. The decision to stop to 2019 was made in accordance to the need of having solid clusters of homogeneous data and relative citations sufficiently consolidated for the articles. We created a dataset, one for each disorder, refining the following parameters: WoS categories (no restrictions), document type (article), language (English), Web of Science Index (Science Citation index expanded – SCIE – or Social sciences Citation index – SSCI). A cluster of 313 275 articles, corresponding to the overall scientific ‘global’ production on seven specific mental health and substance use disorders was obtained. This cluster, made of seven specific datasets, was then exported to ‘InCites’, a citation-based research analytics tool. In InCites we analysed our datasets on the basis of 11 research areas (Psychiatry, Psychology Clinical, Neurosciences, Psychology, Clinical Neurology, Behavioural sciences, substance abuse, neuroimaging, medicine general and internal, psychology applied, psychology psychoanalysis), which correspond to the same 11 categories (or research areas) in WoS and to which mental health topic is associated in SCI-E and SSCI journals. The following parameters were applied to each dataset: dataset (type of disorder), type of analysis (research area), type of document (articles), categories (psychiatry, psychology clinical, neurosciences, psychology, clinical neurology, behavioural sciences, substance abuse, neuroimaging, medicine general and internal, psychology applied, psychology psychoanalysis). In addition, different benchmarks to discern how the information for the performance indicators selected compare against calculated totals were applied [(1) baseline for pinned items, i.e. baseline for all results pinned from the data table; (2) dataset baseline, i.e. enabling the user to benchmark the current custom dataset; dataset baselines are affected by year, document type and research area filter; (3) baseline for all items, i.e. baseline for all items in the results data table; filters are incorporated into the calculation for this baseline]. We downloaded all the articles falling into the baseline for pinned items dataset, obtaining all the publications worldwide. Then, we created a sub-cluster of articles specifically defining a ‘location’ parameter (China, mainland) in InCites, obtaining the overall number of articles authored by researchers affiliated with organisations (institutions) in mainland China (Fig. 2).

### Statistical analysis

Categorical data were described as count and percentages; trends over decades were calculated with nonparametric test for trends across ordered groups (Cuzick, 1985). Average citation is given for each year as the total number of citations divided by the total number of articles published for each disease. A *p* value < 0.05 will be considered significant.

## Results

**Fig. 3.**
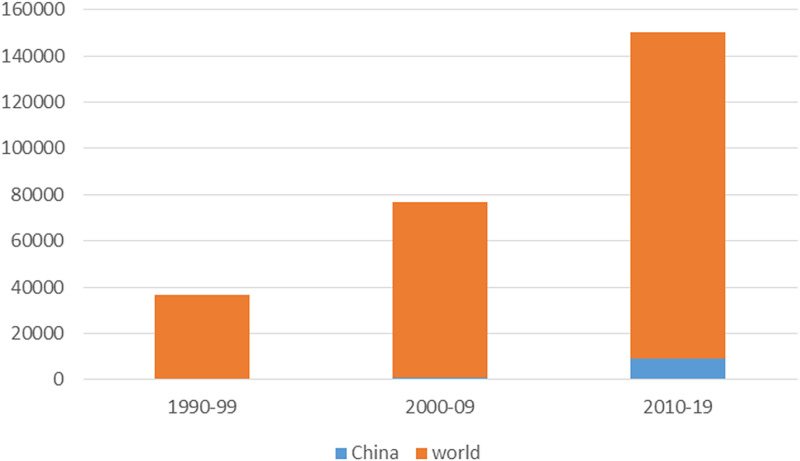
Total volume of published research in seven mental and substance use disorders, world and China.

**Fig. 4.**
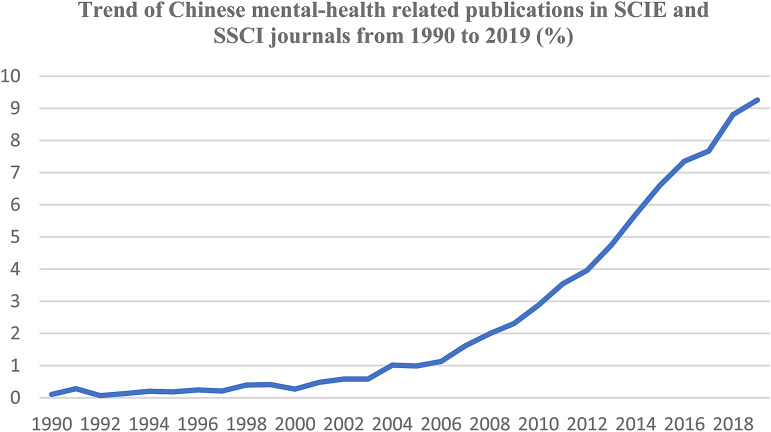
Trend of published research in seven mental and substance use disorders, China.

This bibliometric analysis initially included a cluster of 313 275 published articles worldwide on the seven mental and substance use disorders, as per WoS initial extraction. After performing our analysis in InCites, and eliminating all duplicates, we obtained a total amount of 253 672 articles for the ‘world’ and 9978 for location ‘mainland China’, covering the period from 1990 to 2019. The total volume of published research in mental health, based on seven mental health and substance use disorders, is represented, for China and the world, in Fig. 3. We calculated the number of articles in mental-health produced by Chinese institutions in mainland China out of the total number publications, both as average and as a percentile, and measured the trend of publications on several decades. The trend for Chinese mental-health related publications in SCIE and SSCI journals from 1990 to 2019 (%), is displayed in Fig. 4.

**Fig. 5.**
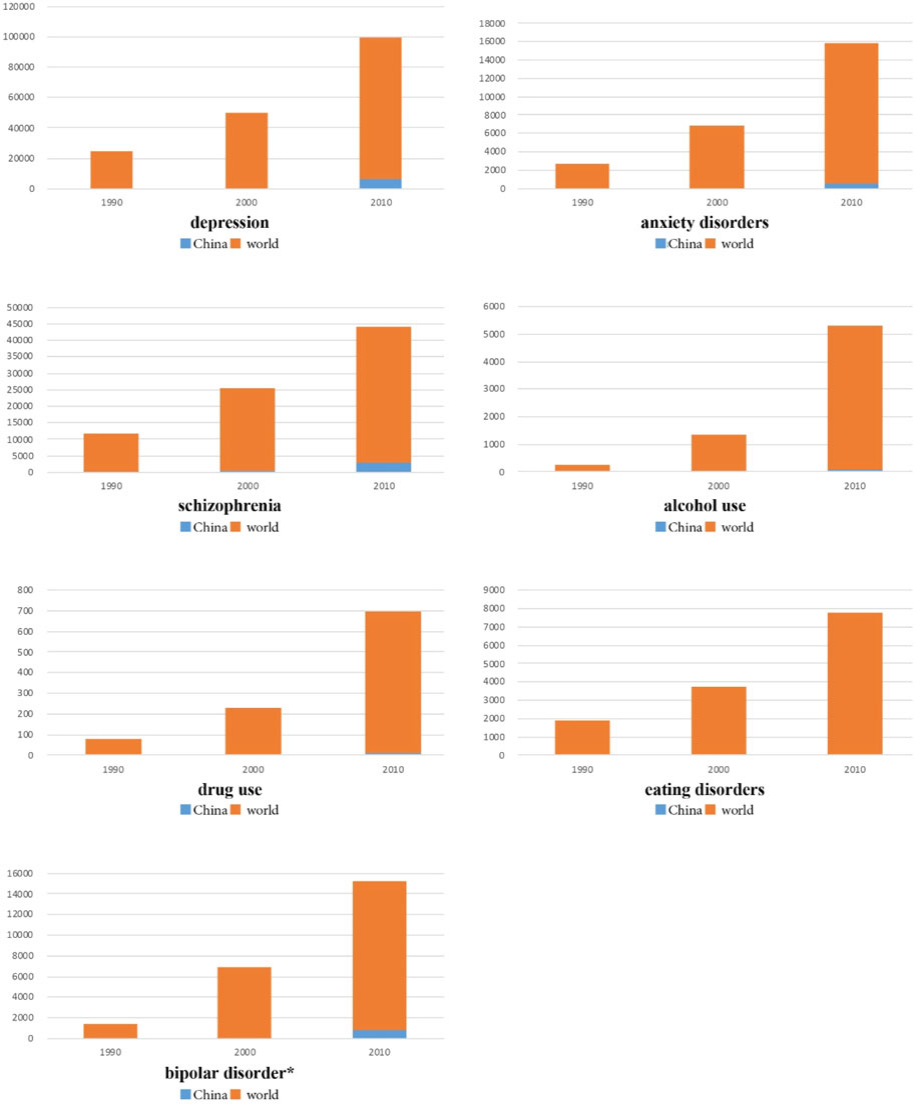
Total number of articles for seven mental and substance use disorder, China and world.

From 1990 to 2020 the overall Chinese scientific production in mental health has been generally increasing, reaching the highest growth in the last decade. Despite the increasing trend, the percentage of articles in mental-health produced by Chinese institutions in mainland China on the overall scientific production worldwide, according to each of the years considered, is below 10%. The total number of articles retrieved for each of the seven mental and substance use disorder is displayed, for China and the world, in the figures below (see Fig. 5).

According to the GBD estimates on prevalence mental and substance use disorders for 1999, 2009 and 2019, the most relevant disorders affecting China are ‘anxiety disorders’, ‘depressive disorders’ and ‘alcohol use’ disorders. In our analysis, a statistically significant increase (*p* < 0.001) is reported for articles produced by Chinese institutions in mainland China regarding ‘depression*’, ‘bipolar disorders*’ and ‘schizophrenia*’. A further analysis on articles in mental-health produced by Chinese institutions in mainland China regarding ‘depression*’, ‘bipolar disorders*’ and ‘schizophrenia*’, was made with respect to the 11 research areas (WoS categories) to which mental health topic is associated in SCI-E and SSCI journals in WoS.

**Fig. 6.**
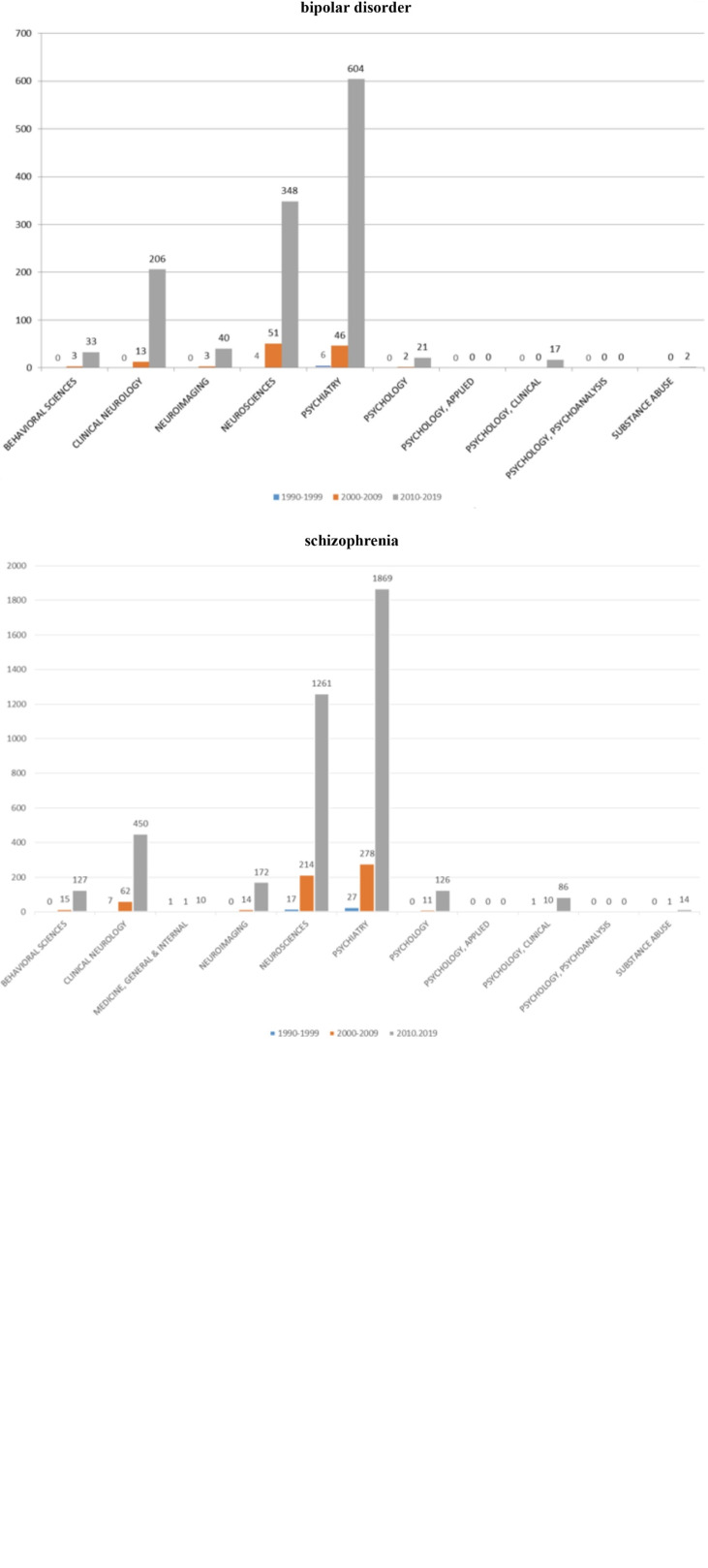
Articles in mental-health produced by Chinese institutions based in mainland China for depression, schizophrenia, bipolar disorders (1990–2019) as per their distribution in 11 research areas associated to mental health in SCIE and SSCI journals.

The analysis provided is limited to ‘depression*’, ‘bipolar disorders*’ and ‘schizophrenia*’ as these three disorders were the only ones presenting a sufficient number of publications to be analysed with respect to the 11 research areas. The results, per different decade, are described below (Fig. 6).

**Fig. 7.**
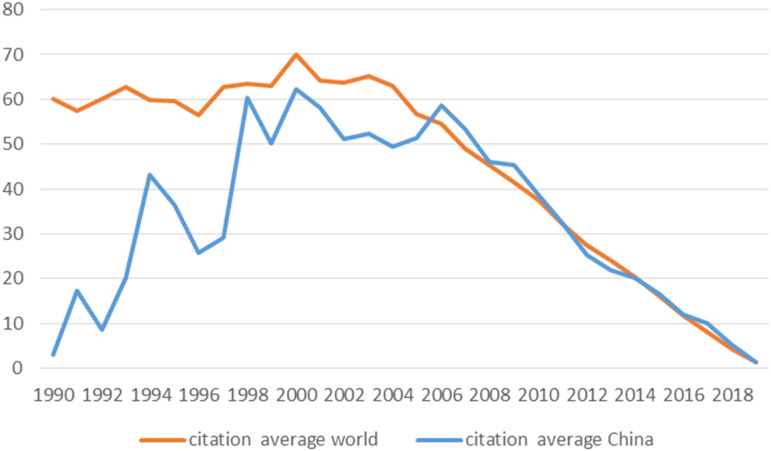
Citation trends for mental-health related publications in SCIE and SSCI journals from 1990 to 2019, world and China.

As mentioned above, among the seven mental and substance use disorders examined, ‘depression*’, ‘bipolar disorders*’ and ‘schizophrenia*’, are representing the highest proportion of articles in mental-health produced by Chinese institutions in mainland China published in international mental-health English journals. This supplementary analysis, including 11 research areas (WoS categories) to which mental health topic is associated in SCI-E and SSCI journals, is evidencing those articles in mental-health produced by Chinese institutions in mainland China are falling predominantly in ‘psychiatry’, ‘neuroscience’ and ‘clinical neurology’ research areas, with a substantial lack of articles falling into ‘psychology, psychoanalysis’, ‘psychology, applied’ and ‘substance abuse’. Published Chinese research is mostly included in SCI-E journals. In order to measure of the impact of Chinese research at the global scientific community level, we analysed the number of citations received by articles in mental-health produced by Chinese institutions in mainland China with respect to the overall number of citations at a global level. To better understand how Chinese mental-health publications fit in the global scientific community, Chinese citations were compared with worldwide citation trends across decades (Fig. 7).

Our analysis is hinting to a substantial overlap, from 2006 onwards, regarding the average number of citations for articles in mental-health produced by Chinese institutions in mainland China and the rest of the world.

## Discussion

This bibliometric analysis showed a rise in the volume of Chinese mental-health related articles in SCI-E and SSCI journals over the past 30 years. At the same rate, the influence of publications from mainland China on international English language publications in mental health has seen an increase in the last period we have analysed. It is worth mentioning that the overlap between the average citations for China and the rest of the world appears to be in line with a very important period in the history of mental health in China. Indeed, a substantial growth in Chinese published research in mental health occurs in the decade 2010–2019, a pivotal period in which extraordinary steps have been taken in direction of a mental health reform in China.

Our results are displaying a statistically significant increase (*p* < 0.001) in the number of mental health related articles produced by Chinese institutions in mainland China concerning ‘depression*’, ‘bipolar disorders*’ and ‘schizophrenia*’ with respect to the other diseases we have analysed. An in-depth investigation of Chinese scientific production through 11 research areas to which mental health topic is associated in SCI-E and SSCI journals, shows that ‘depression*’, ‘bipolar disorders*’ and ‘schizophrenia*’ articles are mainly associated to ‘neuroscience’, ‘psychiatry’ and ‘clinical neurology’ research areas, with a substantial lack of articles falling into ‘psychology, psychoanalysis’, ‘psychology applied’ and ‘substance abuse’ areas.

Our analysis also provides evidence that, despite the increasing trend, the percentage of articles in mental-health produced by Chinese institutions in mainland China on the overall scientific production worldwide is below 10%, thus resulting in a relatively low impact at a global level. A substantial growth in Chinese published research in mental health occurs in the decade 2010–2019, a time during which steps have been taken in direction of a mental health reform in China (The National Mental Health Plan, 2002–2010; Notice to implement National Continuing Management and Intervention Program for psychoses, 2006; The standing conference of State Council of China adopted Guidelines for Furthering the Reform of Health-care System in Principle, 2009; Xiang *et al*., 2012; The National Mental Health Plan, 2015–2020; Xiong and Phillips, 2016; 中国医师协会精神科医师分会, 2019). However, based on the revised literature, several challenges are nowadays affecting the fulfilment of this important reform (Charlson *et al*., 2016; Liang *et al*., 2018; Xiang *et al*., 2018; Huang *et al*., 2019; Que *et al*., 2019).

Furthermore, results are showing that the increment of Chinese publications in ‘depression*’, ‘bipolar disorders*’ and ‘schizophrenia*’ is just partially overlying the burden of most prevalent disorders in China, as per GBD estimates. These findings suggest that Chinese research should be also oriented in achieving an increased proportion of articles pertaining to ‘anxiety disorders*’ and ‘alcohol use disorders*’. In addition, Chinese published mental health research results to be more prolific in articles concerning neurobiological aspects of mental disorders, falling into SCI-E journals. Otherwise, articles deepening psycho-social aspects of mental research and social determinants of mental health are very limited.

### Limitations

The general WoS platform allows to retrieve articles produced in different research areas, but among the 254 available, there is no thematic area specific to mental health. Therefore, we limited our search to seven prevalent mental and substance use disorders worldwide, as entry terms to create our initial cluster of articles in WoS. The WoS Core Collection database serves also as the standard data set underpinning the journals' impact metrics found in the Journal Citation Reports and the institutional performance metrics found in InCites. We used journal-level classification into research areas to indicate disciplines and specialties. The dataset was analysed on the basis of 11 research areas (WoS categories), to which the topic of mental health is associated to SCI-E and SSCI journals indexes in InCites. But in WoS, journals may simultaneously belong to more than one WoS category (Reclassification of papers in Multidisciplinary journals for creating field baselines, 2020) thus resulting in limitations while defining each paper's specific topic and potential misclassification of multidisciplinary research. In InCites, the reclassification process considers this aspect, determining that a given set of documents classified under a specific subject area and other subject areas in WoS are not necessarily mutually exclusive. This results in an article being classified in several categories and the main category is given precisely by this reclassification. It follows that the first category of a paper, the main one, is not always included in those we selected.

## Conclusions

A reflection on the role that China is assuming in the global mental health effort is of a central importance. China's contribution in mental health research is considered to be crucial, and not just in consideration of the country's powerful impact, at environmental, economic and human levels. Great importance is attributed to the trajectories embraced by Chinese mental health research, as a result of its unique historical pathway, practices and experiences. Furthermore, a fundamental interrogation is made around the extent to which this research could impact the global mental health research. This would either provide new insight, or pose new challenges to the existing models. In consideration of the very limited number of studies assessing the collective evidence of Chinese's research in mental health, we developed our analysis with the unique purpose of providing a preliminary picture of the Chinese's scientific input in this field of knowledge. This picture in our opinion is important, for a number of reasons: it gives the trend of Chinese publications in mainland China over a 30-year period and it allows to say that number of Chinese internationally published articles is increasing. Nevertheless, according to this picture, the percentage of articles in mental-health produced by Chinese institutions in mainland China on the overall scientific production worldwide is below 10%. This percentage is low and can ultimately affect the Chinese contribution within the context of global mental health research. Furthermore, our results are showing that the increase in the number of Chinese publications is just partially overlying the burden of most prevalent disorders in China. Also, this internationally published research appears to be more prolific in articles concerning neurobiological aspects of mental disorders. Otherwise, articles deepening psycho-social aspects of mental research and social determinants of mental health are very limited.
